# A Rare Presentation of Xanthogranulomatous Cholecystitis as Bouveret's Syndrome

**DOI:** 10.1155/2012/402768

**Published:** 2012-12-30

**Authors:** Ayşegül Solmaz Tuncer, Safiye Gürel, Zeliha Coşgun, Ahmet Büber, Rıdvan Çakmaz, Oğuz A. Hasdemir

**Affiliations:** ^1^Department of Radiology, Izzet Baysal State Hospital, 14100 Bolu, Turkey; ^2^Department of Radiology, Izzet Baysal School of Medicine, Abant Izzet Baysal University, 14280 Bolu, Turkey; ^3^Department of Radiology, Silifke State Hospital, 33960 Mersin, Turkey; ^4^Department of Radiology, Evliya Celebi Teaching and Research Hospital, 43000 Kutahya, Turkey; ^5^Department of Surgery, University Teaching and Research Hospital, 02200 Adiyaman, Turkey; ^6^Department of Surgery, Diskapi Yildirim Beyazit Teaching and Research Hospital, 06000 Ankara, Turkey

## Abstract

The purpose of this paper is to present sonographic and CT imaging findings of xanthogranulomatous cholecystitis (XGC) presented as Bouveret's syndrome, a very rare cause of gastric obstruction. While the patient's physical examination, upper GI endoscopy, and radiological findings all pointed to Bouveret's syndrome, CT differential diagnosis suggested either XGC or gallbladder carcinoma, and the final diagnosis was done histopathologically. Our paper aims to increase awareness in radiologically diagnosing XGC cases by introducing the possibility of existence of Bouveret's syndrome.

## 1. Introduction

Xanthogranulomatous cholecystitis (XGC) is an unusual disease, with an estimated incidence of between 0.7% to 13.2% of all cholecystitis cases, while the frequency is about 1.5% of all the cholecystectomies studied [1]. Cholecystolithiasis incidence rate in most reports is 85% to 100% [2, 3]. The clinical presentation of XGC resembles acute or chronic cholecystitis, and it is not uncommon to have a difficult cholecystectomy and it may be difficult to differentiate it from gallbladder (GB) cancer until the pathologic specimen is examined [1]. Complications include perforation, abscess formation, fistulous tracts, and extension of the inflammatory process neighbouring structures [4].

Bouveret's syndrome, on the other hand, is also known as “gastric outlet obstruction” and is a rare condition representing fewer than 5% of the cases with gallstone ileus, which itself is a complication in only 0.3–4% of cases having cholecystolithiasis [5]. 

Despite a few studies with small series in the literature, there is a lack of data about the exact prevalence of the Bouveret's syndrome accompanying XGC [6]. In this paper we present ultrasonography (US) and computed tomography (CT) findings of a xanthogranulomatous cholecystitis presented as Bouveret's syndrome.

## 2. Case Report

**Figure 1:**
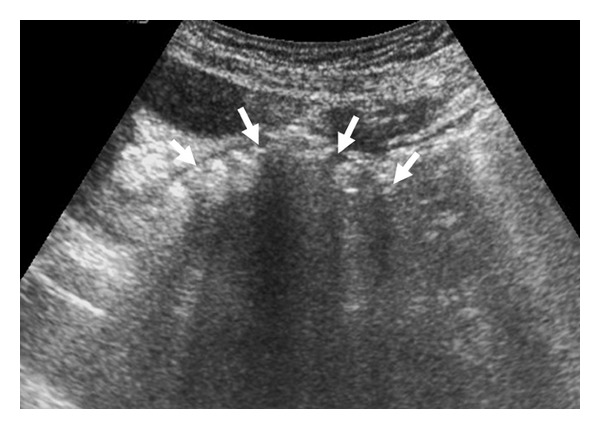
A gray-scale trans abdominal US image obtained at axial plane shows a mass (arrows) within duodenum having a smooth and curvilinear surface, and causing posterior acoustic shadowing.

**Figure 2:**
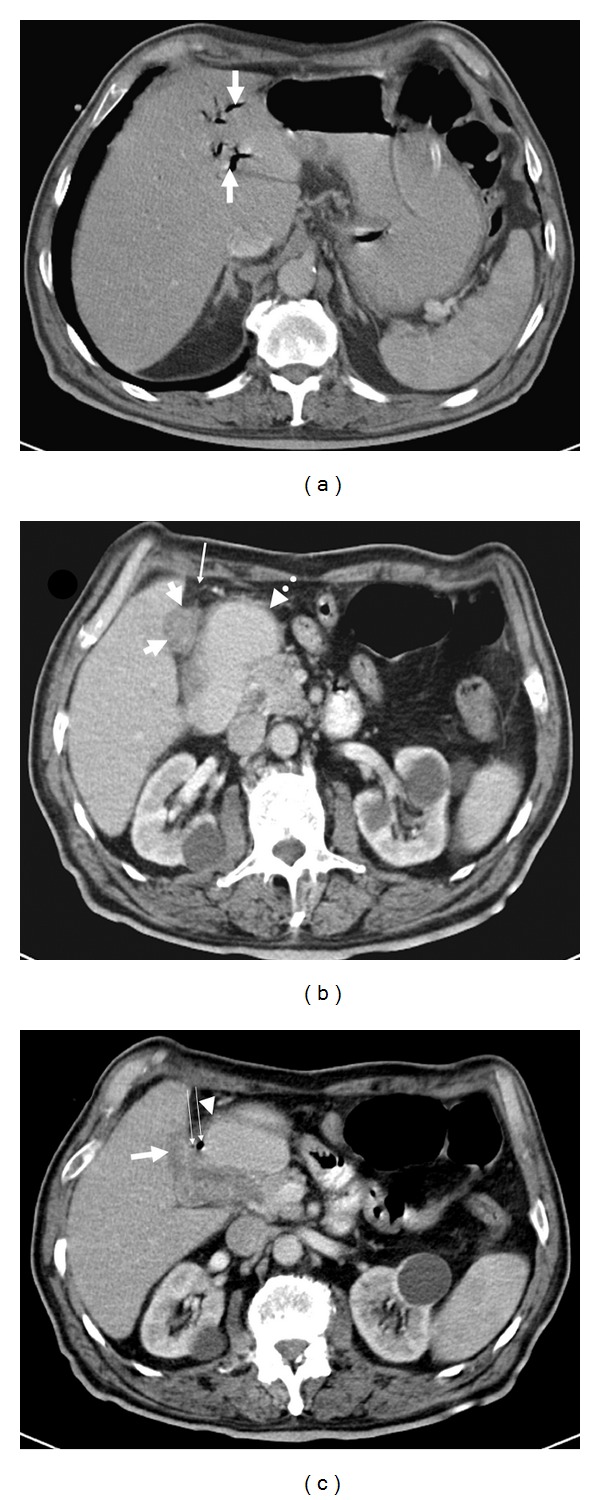
(a) On nonenhanced axial CT image, air in the biliary tree of the left lobe is illustrated. A nasogastric tube within dilated stomach is noted as well. (b) On post-contrast portal venous phase, axial CT image, a collapsed GB (short arrows) having irregularly thickened, heterogeneously hypoechoic, and slightly enhancing wall accompanied with pericholecystic stranding (long arrow) and the dilatation of first and second portions of duodenum (dotted arrow) are illustrated. (c) On post-contrast portal venous phase axial CT image, irregularly thickened and heterogenous GB wall (short arrow) is imperceptible from the medial duodenal wall (arrowhead) and at this area, two air bubbles are identified (long arrows) indicating a cholecystoduodenal fistula.

**Figure 3:**
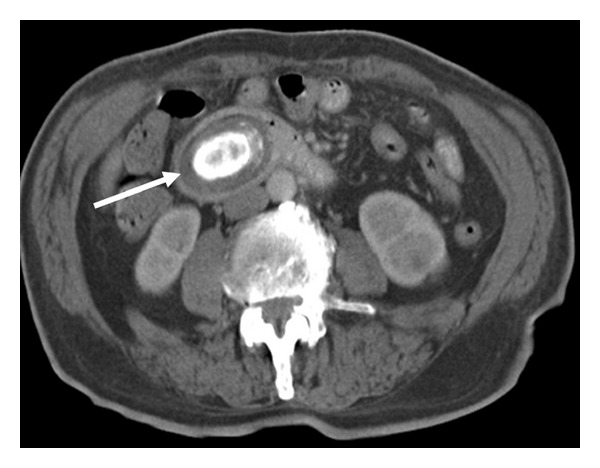
On post-contrast axial CT image a giant gallstone is seen occupying the 3rd and 4th portions of the duodenum.

A 75-year-old man was admitted to emergency department with a 20-day history of worsening nausea, vomiting, hiccupping, obstipation, and inability to pass flatus. He had cachexia associated with a remarkable decrease in turgor and tonus. Physical examination demonstrated tenderness in the right upper quadrant. US was suboptimal due to the massive intestinal gas and uncooperative nature of the patient. A collapsed GB and a mass with a smooth curvilinear surface and posterior acoustic shadowing in the distal part of the duodenum were the only distinguishable findings (Figure 1). After an upper gastrointestinal endoscopy, which was inconclusive due to massive bile in the duodenum, the patient underwent an abdominal CT examination. On nonenhanced axial CT image pneumobilia was seen in the left lobe of the liver and gastric dilatation was seen (Figure 2(a)); on the post-contrast images was obtained at portal venous phase, duodenal dilatation, decompressed GB with an irregularly thickened and heterogeneous and slightly enhancing wall with minimal pericholecystic stranding were detected (Figure 2(b)). Walls of the gallbladder and the duodenum were not discernible from each other and there was free air at that region which pointed out a cholecystoduodenal fistula in the presence of pneumobilia (Figure 2(c)). Additionally, gastric and duodenal dilatation and a giant (7 × 8 × 5.5 cm) gallstone occupying the 3rd and 4th portions of the duodenum were detected on CT (Figure 3). Due to the persisting upper gastrointestinal tract obstruction, surgery was planned on the 7th day from the admission. 

Intraoperatively, decompressed and edematous GB was seen to be fistulized to the second part of the duodenum. Duodenal and proximal jejunal loops were dilated. The gallstone causing obstruction was found in the last segment of the dilated proximal jejunal loops. Cholecystectomy and primary repair of the duodenum was performed and followed by the placement of an 8 French T-tube in the common bile duct. The gallstone was extracted from the jejunum with a simple enterotomy made 25 cm distal to the ligamentum Treitz followed by an antecolic gastroenterostomy. Histopathological examination of the GB came out to be xanthogranulomatous cholecystitis.

## 3. Discussion

XGC is an unusual form of chronic cholecystitis and predominantly seen in women between the ages of 60 and 70 years. Patients normally present with signs and symptoms of cholecystitis: right upper quadrant pain, vomiting, leukocytosis, and a positive Murphy sign. Slightly less than one-half of patients have a tender, palpable, right upper quadrant mass at physical examination. Some patients present with anorexia [3, 7]. Our patient was a man who had cachexia and a history of worsening nausea and vomiting, and tenderness at the right upper quadrant. However, neither findings on physical examination nor results of laboratory tests appeared to be useful in the differential diagnosis of XGC among other more frequent cholecystitis types or GB carcinoma. Although the mechanism leading to the formation of XGC has not been firmly established, the extravasation of bile into the gallbladder wall is believed to have a role in the development of the inflammatory process and XGC is often mistaken for GB carcinoma. Nevertheless a positive association between XGC and carcinoma of the GB has been reported and is explained as the development of metaplastic and dysplastic changes due to inflammatory phenomena [3, 7]. 

On US, XGC may appear as a moderate-to-marked thickening of the GB wall and hypoechoic bands or nodules within the thickened gallbladder wall. Other sonographic findings include disruption of the mucosal line, pericholecystic fluid, stones, and intrahepatic biliary dilatation. However, due to the decompression of the GB, US might have difficulty in showing the changes in the wall as in our patient. Clean or dirty acoustic shadowing from stones or air within the GB lumen, air in the bile ducts, dilatation of small bowel, and fistula can be detected sonographically [8]. The only sonographic findings in our patient were a collapsed GB and a mass like structure causing posterior acoustic shadowing in the duodenum. We failed to detect either the fistula or the air in the biliary tree due to the massive intestinal gas and uncooperative patient. 

CT may demonstrate low-attenuation foci in the gallbladder wall that corresponds to the hypoechoic nodules seen at US with an irregular, sometimes lobulated, and greatly thickened wall in XGC. The GB mucosa typically enhances with contrast material. CT more effectively demonstrates adjacent organ involvement and infiltration of the adjacent soft tissue and fat planes when compared with US. Occasionally, biliary dilatation is present and may be secondary to the presence of intraductal stones, hepatoduodenal ligament adenopathy, or in rare cases a coexistent malignancy of the gallbladder or bile duct. The preoperative diagnosis of XGC is also difficult with CT and most of the patients were diagnosed by an intraoperative frozen section examination or postoperative specimen [3, 6, 7]. Although the GB was decompressed in our patient, there were prominent heterogeneous hypodensity, irregular thickening and minimal heterogeneous enhancement of the wall with minimal pericholecystic stranding indicating either GB carcinoma or xanthogranulomatous cholecystitis which both might cause cholecystoduodenal fistula.

Bouveret's syndrome is frequently preceded by one or several episodes of acute cholecystitis resulting inflammation and adhesions facilitating the erosion of the gallstone through the gallbladder wall forming a cholecystoenteric fistula. The fistula can be cholecystoduodenal (60%), cholecystocolic (17%), cholecystogastric (5%), choledochoduodenal (5%) [9]. Complications of XGC are present in 32% of cases including perforation, abscess formation, fistulous tracts to the duodenum or skin, and extension of the inflammatory process to the liver, colon, or surrounding soft tissues [4]. The incidence of a gallbladder fistula to stomach, colon, common bile duct and duodenum were reported about 13 percent with XGC [2, 4]. To our knowledge, presentation of the XGC as gallstone ileus such as in our patient is rare in the current literature [6].

CT can identify stones in the intestinal lumen, intestinal obstruction, pneumobilia, and sometimes the bilioenteric fistula in the Bouveret's syndrome. In our patient, who had no prior ERCP or surgery, the abdominal CT demonstrated pneumobilia, a big gallstone within the last two portions of the duodenum, and gastric dilatation. Bilioenteric fistula can be identified with different modalities such as an upper gastrointestinal barium study, CT, ERCP, MRCP [8, 10]. In our patient, the cholecystoduodenal fistula was depicted as a free-air containing area where the thickened duodenal and the gallbladder wall were indistinguishable from each other. However, neither CT findings of GB wall nor the presence of gallstone ileus were specific enough to exclude GB carcinoma. Both XGC and GB carcinoma might accompany gallstones and/or cholecystoduodenal fistula, therefore the definite diagnosis was histopathological in our patient.

In conclusion, although the formation of firm adhesions and fistulas are not unusual in the natural course of XGC because of destructive inflammation, to the best of our knowledge, the presentation of the XGC as gallstone ileus as in our patient has not been demonstrated in the current literature. We believe the combination of these two entities can be an emergency in both clinical and radiological aspects and is a topic that all radiologists should be aware of.
